# Glucose transporter 1 expression as a marker of prognosis in oesophageal adenocarcinoma

**DOI:** 10.18632/oncotarget.24906

**Published:** 2018-04-06

**Authors:** Jaine K. Blayney, Lauren Cairns, Gerald Li, Niamh McCabe, Leanne Stevenson, Christopher J. Peters, Nathan B. Reid, Veronica J. Spence, Chintapuza Chisambo, Damian McManus, Jacqueline James, Stephen McQuaid, Stephanie Craig, Kenneth Arthur, Darragh McArt, Chin-Ann J. Ong, Pierre Lao-Sirieix, Peter Hamilton, Manuel Salto-Tellez, Martin Eatock, Helen G. Coleman, Rebecca C. Fitzgerald, Richard D. Kennedy, Richard C. Turkington, On behalf of the Oesophageal Cancer Clinical and Molecular Stratification (OCCAMS) Study Group

**Affiliations:** ^1^ Centre for Cancer Research and Cell Biology, Queen's University Belfast, Belfast, Northern Ireland, UK; ^2^ Department of Surgery and Cancer, Imperial College, London, UK; ^3^ Northern Ireland Molecular Pathology Laboratory, Centre for Cancer Research and Cell Biology, Queen's University Belfast, Belfast, Northern Ireland, UK; ^4^ Hutchison/MRC Cancer Unit, University of Cambridge, Cambridge, UK; ^5^ Northern Ireland Cancer Centre, Belfast City Hospital, Lisburn Road, Belfast, Northern Ireland, UK; ^6^ Centre for Public Health, Queen's University Belfast, Belfast, Northern Ireland, UK

**Keywords:** oesophageal cancer, glucose transporter 1, hypoxia, prognostic, biomarker

## Abstract

**Background:**

The current TNM staging system for oesophageal adenocarcinoma (OAC) has limited ability to stratify patients and inform clinical management following neo-adjuvant chemotherapy and surgery.

**Results:**

Functional genomic analysis of the gene expression data using Gene Set Enrichment Analysis (GSEA) identified GLUT1 as putative prognostic marker in OAC.

In the discovery cohort GLUT1 positivity was observed in 114 patients (80.9%) and was associated with poor overall survival (HR 2.08, 95% CI 1.1-3.94; p=0.024) following multivariate analysis. A prognostic model incorporating GLUT1, CRM and nodal status stratified patients into good, intermediate and poor prognosis groups (p< 0.001) with a median overall survival of 16.6 months in the poorest group.

In the validation set 182 patients (69.5%) were GLUT1 positive and the prognostic model separated patients treated with neo-adjuvant chemotherapy and surgery (p<0.001) and surgery alone (p<0.001) into three prognostic groups.

**Patients and Methods:**

Transcriptional profiling of 60 formalin fixed paraffin-embedded (FFPE) biopsies was performed. GLUT1 immunohistochemical staining was assessed in a discovery cohort of 141 FFPE OAC samples treated with neo-adjuvant chemotherapy and surgery at the Northern Ireland Cancer Centre from 2004-2012. Validation was performed in 262 oesophageal adenocarcinomas collected at four OCCAMS consortium centres. The relationship between GLUT1 staining, T stage, N stage, lymphovascular invasion and circumferential resection margin (CRM) status was assessed and a prognostic model developed using Cox Proportional Hazards.

**Conclusions:**

GLUT1 staining combined with CRM and nodal status identifies a poor prognosis sub-group of OAC patients and is a novel prognostic marker following potentially curative surgical resection.

## INTRODUCTION

In Western populations the incidence of oesophageal adenocarcinoma (OAC) in men has risen by 6-fold in the last 30 years and has become the dominant histological subtype [1, 2]. The addition of neo-adjuvant therapy prior to surgical resection has improved survival for localised disease but there are wide variations in outcomes [3, 4].

The prediction of prognosis after potentially curative surgery is currently based on the internationally accepted TNM classification system but its limited ability to stratify patients has led to the investigation of additional biomarkers. Pathological features, such as nodal involvement, lymphovascular invasion (LVI) and circumferential resection margin (CRM) status, provide additional prognostic information and in cases treated with neo-adjuvant chemotherapy a histopathological response in the tumour or lymph nodes has been shown to predict improved survival [5–7]. However, a recent analysis of the Medical Research Council Gastric Infusional Chemotherapy (MAGIC) trial demonstrated that positive lymph node status and not pathological response was the only independent predictor of survival, albeit in a predominantly gastric adenocarcinoma sample set (76%) [8]. The use of 2-[18F]fluoro-2-deoxy-D-glucose (FDG) positron emission tomography (PET) to predict benefit to chemotherapy and prognosis has also been investigated with OACs displaying persistently high levels of FDG uptake, even after the administration of chemotherapy, associated with higher rates of relapse and poor survival [9, 10]. Current prognostic strategies do not take into account the molecular features of each tumour which may explain the wide variation in survival amongst patients with similar metabolic and pathological staging. Robust prognostic biomarkers are required to enable the stratification of OAC patients following surgical resection.

Recently, comprehensive whole genome sequencing and multiple platform analyses have led to the discovery of novel molecular sub-groups in OAC and may improve prognostication [11–14]. At present the application of next-generation sequencing techniques has not extended to routine use in pathology laboratories and so there remains a role for immunohistochemical (IHC) biomarkers. A wide range of IHC prognostic markers have been investigated in OAC, including the development and validation of a three gene prognostic panel of Epidermal Growth Factor (EGFR), Tripartite Motif-containing 44 (TRIM44) and Sirtuin 2 (SIRT2), but to date none have entered routine clinical practice [15–17]. Markers may be discovered through an understanding of the molecular biology of OAC, by analysis of genomic data or from being identified as a prognostic marker in other tumour types. Once identified the studies for each marker are often limited by a small number of cases, absence of a defined scoring methodology and lack of validation in an independent dataset.

In the current study we, therefore, aimed to identify robust IHC prognostic markers which could be used to inform the management of OAC using FFPE material. Specifically, we focused on the identification of potential biomarkers from transcriptional data followed by development and validation of an IHC marker. Unlike previous studies, we examined the interaction of our biomarkers with pathological features and have developed a prognostic model which could be used in post-chemotherapy resection material to inform post-operative surveillance or treatment strategies.

## RESULTS

### Gene expression and gene set enrichment analysis (GSEA)

Functional analysis of gene expression data from 60 FFPE OAC biopsies (Supplementary Table 1) was performed to generate a list of candidate prognostic biomarkers. Cases were divided into pathological responders (TRG ≤2, n=7) and non-responders (TRG 3-5, n=53) and GSEA using the C2 canonical pathways gene set database identified ten pathways associated with pathological response to neo-adjuvant chemotherapy (Supplementary Table 2). The top-ranked pathway was the Hypoxia-inducible factor 1 (HIF1) pathway and a ranked gene list of HIF1 pathway genes, beginning with the most upregulated gene in the non-responders compared to the responders was generated (Supplementary Figure 1, Supplementary Table 3). The enriched genes from this pathway contained a number of genes implicated as prognostic markers in oesophageal and other cancers, such as Insulin-like Growth Factor Binding Protein 1 (IGFBP1), N-myc Downstream Regulated Gene 1 (NDRG1), Trefoil Factor 3 (TFF3) and Solute Carrier Family 2 Member 1/Glucose Transporter 1 (SLC2A1).

### Candidate gene selection

To select a candidate biomarker from the HIF1 pathway an MA plot of gene expression levels corresponding to the HIF1 pathway was performed and a False Discovery Rate (FDR) of <0.1 applied (Supplementary Figure 2). We further assessed the list of candidates according to the published literature for genes with a biological role in oesophageal cancer, potential as a prognostic marker and for availability of a validated antibody. Four candidates were selected (SLC2A1, IGFBP1, NDRG1, TFF3) of which SLC2A1 was the only gene to have FDR <0.1 and expression of SLC2A1 was strongly correlated with both IGFBP1 and NDRG1 (Spearman's correlation co-efficient; FDR <0.1) indicating its expression was representative of these HIF1 pathway candidates. SLC2A1 encodes the membrane protein Glucose Transporter 1 (GLUT1) and high expression of GLUT1 has been associated with poor survival in oesophageal squamous cell carcinoma and progression of Barrett's oesophagus to adenocarcinoma but GLUT1 has not been studied as a prognostic marker in OAC [18–23]. The primary mechanism for the accumulation of FDG in cancer cells during PET scans is through GLUT1-mediated transport and so the persistently high levels of FDG uptake associated with a poor prognosis in OAC may be mediated by high GLUT1 expression [24, 25]. Considering the prognostic ability of GLUT1 in a range of tumour types, its role in PET response and the availability of a robust and validated antibody we selected GLUT1 for further investigation as a prognostic marker in OAC.

### GLUT1 immunohistochemistry

**Figure 1:**
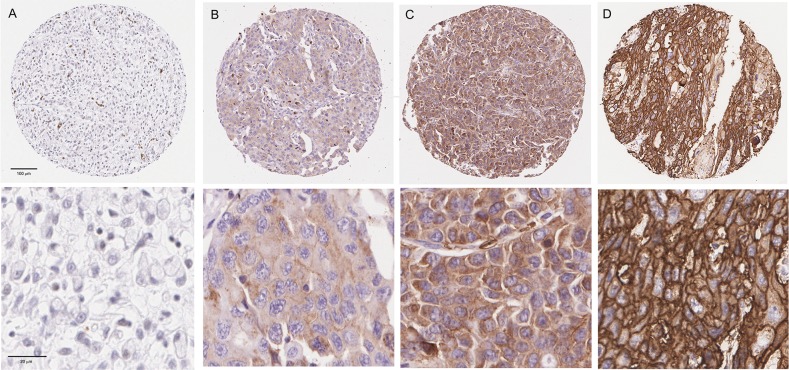
Tissue Microarray GLUT1 staining Representative 10X and 40X views of tumours showing negativity for GLUT1 **(A)** and weak **(B)**, moderate **(C)** and strong **(D)** staining for GLUT1.

Expression of GLUT1 was observed in the cytoplasm and at the cell membrane of OAC cells and heterogeneous staining was noted. (Supplementary Figure 3) [24]. GLUT1 expression was assessed in a discovery set consisting of resection specimens from 141 oesophageal and gastro-oesophageal junction adenocarcinoma patients (Table 1) and no GLUT1 staining was observed in 27 cases (19.1%) with weak, moderate and strong staining observed in 49 (34.8%), 37 (26.2%) and 29 (20.6%) cases respectively (Figure 1). Close agreement was observed between three independent pathologists (Concordance correlation coefficient= 0.88; 95% CI 0.83-0.91). No significant association was found between GLUT1 and expression of the clinically validated markers Her2, p53 or MET (Supplementary Table 4).

**Table 1:** Comparison of discovery and validation tissue microarrays

	Discovery TMA NICC	Validation TMA OCCAMS	*p*-value
	*n* = 141 (%)	*n* = 262 (%)	
**Age**			
<60	42 (30)	80 (31)	0.034
60-69	67 (47)	92 (35)	
≥ 70	32 (24)	86 (33)	
Unknown	0	4 (2)	
Median	63	66	0.058^†^
Range	28-83	33-88	
**Sex**			
Male	110 (78)	213 (81)	0.431
Female	31 (22)	49 (19)	
Unknown	0	6 (2)	
**Tumour Site**			
Oesophagus	22 (17)	262 (100)	NA
GOJ, Siewert 1	72 (52)		
GOJ, Siewert 2	35 (23)		
GOJ, Siewert 3	12 (8)		
**Depth of Invasion (T stage)**			
pT0/1	15 (11)	21 (8)	0.578
pT2	27 (19)	56 (21)	
pT3	94 (67)	180 (69)	
pT4	5 (4)	5 (2)	
**Lymph node Involvement (N stage)**			
N0	51 (36)	72 (27)	0.008
N1	29 (21)	92 (35)	
N2/3	61 (43)	96 (37)	
Unknown	0	2 (1)	
**Differentiation**			
Well	6 (4)	21 (8)	0.189
Moderate	53 (38)	79 (30)	
Poor	81 (57)	151 (58)	
Unknown	1 (1)	11 (4)	
**Lymphovascular Invasion**			
Negative	47 (33)	98 (37)	0.004
Positive	93 (66)	101 (39)	
Unknown	1 (1)	63 (24)	
**Circumferential Margin Involvement**			
Negative	77 (55)	113 (43)	0.073
Positive	63 (45)	61 (23)	
Unknown	1 (1)	88 (34)	
**Neo-Adjuvant chemotherapy**			
Yes	141 (100)	127 (48)	<0.0001
No	0	135 (52)	

^†^Mann-Whitney *U* Test

NICC- Northern Ireland Cancer Centre

OCCAMS- Oesophageal Cancer Clinical and Molecular Stratification Consortium.

To increase the clinical utility of GLUT1 as a biomarker, and to enable a more reliable scoring methodology, we defined GLUT1 positivity as any cancer cells staining for GLUT1, regardless of the intensity or percentage of cells. With the exception of tumour differentiation, there were no significant differences in any major clinicopathological factors between GLUT1 negative and positive patients (Supplementary Table 5).

The relationship between GLUT1 expression, four known pathological factors (LVI, CRM, differentiation and nodal status) and prognosis were examined further by univariate (Supplementary Table 6) and multivariate analysis (Table 2). In univariate analysis GLUT1 positivity was a significant predictor of reduced relapse-free survival (RFS) (HR 2.07, 95% CI 1.09-3.93; p=0.026) with a median RFS of 21.2 (95% CI 15.9-29.2) and 63.7 (95%CI 28.1-NA) months for GLUT1 positive and negative patients respectively (Supplementary Figure 4A). Overall survival (OS) was also significantly reduced for GLUT1 positive patients (HR 1.85, 95% CI 1.01-3.39; p=0.047) with a median OS of 31.6 (95%CI 23.4-38.4) months in GLUT1 positive patients and 43.9 (95%CI 32.7-NA) months in GLUT1 negative cases (Supplementary Figure 4B). Following multivariate analysis GLUT1 positivity remained an independent prognostic factor alongside CRM and nodal status in this cohort uniformly treated with neo-adjuvant chemotherapy and surgical resection (Table 2). Variables were selected using the elastic net penalty method (Supplementary Table 7) and the concordance index to assess the predictive fit of each model (Supplementary Table 8). The relative contribution of each variable was also assessed by the log likelihood ratio, with the greatest effect observed for nodal status (Supplementary Table 9).

**Table 2:** Multivariate analysis of clinicopathological factors, GLUT1 expression, relapse-free and overall survival in the discovery cohort

	Relapse-free Survival			Overall Survival		
	Hazard Ratio	95% CI	*p*-value	Hazard Ratio	95% CI	*p* -value
**CRM**						
Negative	1			1		
Positive	2.059	1.167-3.631	0.01	1.897	1.067-3.373	0.03
**N Stage**						
0	1			1		
1	1.542	0.749-3.176	0.24	2.189	0.999-4.796	0.05
2/3	3.962	2.017-7.782	<0.001	5.694	2.742-11.823	<0.001
**Differentiation**						
Poor	1			1		
Moderate	1.19	0.717-1.967	0.502	1.05	0.194-3.703	0.827
Well	0.862	0.198-3.763	0.844	0.849	0.624-1.764	0.855
**GLUT1**						
Low	1			1		
High	2.352	1.191-4.647	0.014	2.059	1.08-3.922	0.028

**Figure 2:**
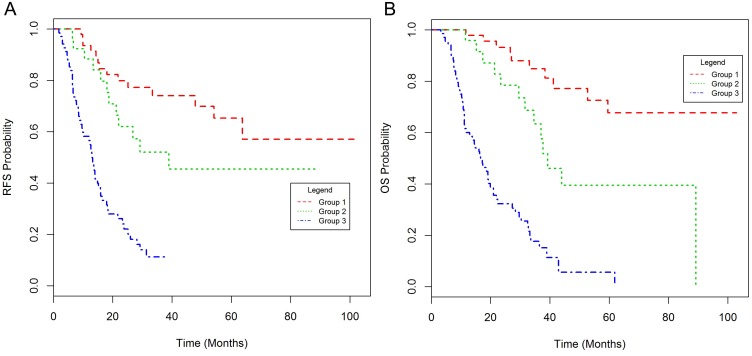
Kaplan-Meier plots of relapse-free **(A)** and overall survival **(B)** for the prognostic score in the discovery set.

Probability nomograms for RFS and OS (Supplementary Figure 5 and Table 2) combining GLUT1, CRM and nodal status were derived from the respective multivariate Cox regression models. Prognostic indices for OS and RFS were developed from the respective nomograms, based on combinations of GLUT1, CRM and nodal status. Each prognostic index was used to separate patients into three groups - good, intermediate and poor prognosis (Table 3). The poor prognosis group was associated with an RFS and OS of 13 and 16.6 months respectively following surgical resection (Figure 2).

**Table 3:** Prognostic model incorporating N stage, CRM and GLUT1 in the discovery cohort

Prognostic Group	Variable Combination		Median RFS (95% CI months)	Median OS (95% CI months)
	N Stage	GLUT1/CRM Status		
**Group 1**	N Stage 0	GLUT1 Negative AND CRM Negative	Not reached	Not reached
	N Stage 1			
**Group 2**	N Stage 2/3	GLUT1 Negative AND CRM Negative	39 (21.2-NA)	39.2 (34.5-NA)
	N Stage 0	GLUT1 Positive AND CRM Positive		
	N Stage 1	GLUT1 Positive OR CRM Positive		
**Group 3**	N Stage 1	GLUT1 Positive AND CRM Positive	13 (9.9-15.9)	16.6 (11.7-20.9)
	N Stage 2/3	GLUT1 Positive AND/OR CRM Positive		

### Validation cohort

**Figure 3:**
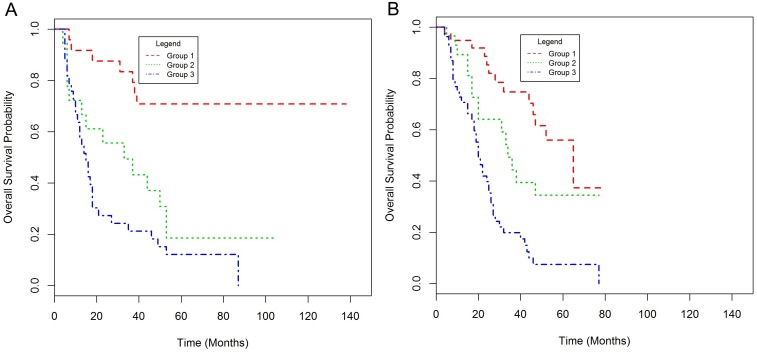
Kaplan-Meier plots of overall survival for the prognostic score in patients treated with neo-adjuvant chemotherapy and surgery **(A)** and those treated with surgery alone **(B)** in the validation set.

The validation set consisted of 262 OAC patients of whom 127 (69.8%) were treated with neo-adjuvant chemotherapy followed by surgery and 135 (74.2%) by surgery alone (Table 1, Supplementary Table 10). Patients in the validation set were older, had a significantly higher nodal status and a lower proportion of cases were LVI positive. GLUT1 expression did not show a significant correlation with any pathological factor but higher GLUT1 positivity was observed in chemotherapy-naïve patients (Supplementary Table 11). GLUT1 positive patients had a significantly worse prognosis (HR 1.85, 95% CI 1.11-3.08, p = 0.018) with a median OS of 18 (95% CI 17-24) and 39 (95% CI 33-53) months in GLUT1 positive and negative patients respectively (Supplementary Figure 6). GLUT1 was confirmed as a statistically significant prognostic indicator of worse OS in both the univariate and multivariate analysis for the neo-adjuvant chemotherapy and surgery treated patients but did not retain significance in the multivariate analysis for the chemotherapy-naïve patients (Supplementary Table 12). However, the prognostic model developed in the NICC cohort successfully stratified patients into three prognostic groups regardless of whether the patient received neo-adjuvant chemotherapy or not (p<0.001) (Figure 3, Supplementary Table 13).

These results confirm the ability of our prognostic score incorporating GLUT1, CRM and nodal status to stratify patients following oesophagectomy and, in particular, to identify a poor prognosis group of patients.

## DISCUSSION

In this study we have performed biomarker selection using gene expression data and identified GLUT1 as an immunohistochemical marker. We have developed a prognostic model incorporating GLUT1 expression, CRM and nodal status which stratifies patients into three prognostic groups when applied to OAC resection material in two independent cohorts.

It has long been recognized that the upregulation of glycolysis, known as the Warburg effect, is a characteristic feature of cancer cells [26]. Intra-tumoural hypoxia leads to increased glycolytic enzyme activity in many cancer types and in response cancer cells upregulate GLUT1, under the control of HIF-1α [26, 27]. The presence of a high degree of intra-tumoural hypoxia has been correlated with invasion and metastases of malignant tumours and may promote refractoriness to anti-cancer therapies [28–30]. The heterogeneous staining of GLUT1 observed in our study is in keeping with evidence suggesting that GLUT1 is expressed primarily in hypoxic areas of the tumour [31].

Strengths of our study include the definition of GLUT1 positivity as any tumour cells staining for GLUT1, increasing the clinical applicability of the assay. The development of a prognostic model increased the ability to stratify patients compared to GLUT1 status alone and validation in an independent sample set was a further strength. Due to the greater age of the samples, a proportion of patients in the validation set did not receive neo-adjuvant chemotherapy and this may account for the differing levels of GLUT1 positivity in the discovery (80.9%) and validation (69.5%) sets as chemo-sensitive tumours which demonstrated a pathological response will not be represented on the discovery TMA. The finding of a significantly lower proportion of N stage 0 patients in the validation cohort may also reflect insufficient pre-operative staging prior to the introduction of PET scans and the reduced administration of neo-adjuvant chemotherapy. In keeping with its discovery in a neo-adjuvant chemotherapy treated cohort, GLUT1 expression predicted survival in the validation set in the context of neo-adjuvant chemotherapy and surgery but not with surgery alone. However, the prognostic model successfully stratified patients in both settings indicating its robustness.

Limitations of our study included the use of pre-chemotherapy biopsy material for the discovery of candidate biomarkers by gene expression analysis followed by the investigation of their protein expression in post-chemotherapy resection material. It was not possible to examine the expression of GLUT1 in the biopsies due to the limited amount of tumour tissue available and the assessment of markers of poor prognosis in post-chemotherapy tissue is consistent with current data indicating the persistence of oncogenic drivers which contribute to prognosis throughout neo-adjuvant chemotherapy [32]. Therefore, we surmised that a dominant marker in a poor prognosis subgroup would be consistently detected in biopsy material and residual tumour tissue which did not respond to chemotherapy.

Our work establishes a role for GLUT1 in more accurate prognostication for patients and clinicians. When combined with nodal status and CRM into a prognostic model GLUT1 contributes to the separation of patients into three prognostic groups. The poorest prognostic group has a median OS of 16.6 (95% CI 11.7-20.9) and 20 (95% CI 18-26) months in the discovery and validation sets respectively, calling into question the validity of treating these patients with the high morbidity approach of neo-adjuvant chemotherapy and surgery. It is tempting to suggest that patients identified as having a higher risk of recurrence should be selected for more intensive follow-up or receive alternative post-operative treatment. Whilst intensive surveillance regimes have been shown to detect cases of local recurrence following radical chemo-radiotherapy suitable for salvage oesophagectomy the role of such follow-up strategies following surgical resection for OAC remains controversial [33]. Considering its role in tumour progression and resistance to therapy, GLUT1, and intra-tumoural hypoxia in general, may also represent a potential adjuvant therapeutic target [34].

In conclusion we have identified GLUT1 expression as a potential biomarker for poor prognosis in resected OAC. We have established a promising assay incorporating IHC and pathological factors which is informative for patient care following chemotherapy and surgical resection.

## PATIENTS AND METHODS

This study was performed and reported in line with the REMARK recommendations (Supplementary Figure 7 and Supplementary Table 14) [35, 36].

### Gene expression profiling from FFPE tissue

Transcriptional profiling of sixty pre-treatment endoscopic OAC FFPE biopsies from patients treated with neo-adjuvant chemotherapy followed by surgical resection at the Northern Ireland Cancer Centre (NICC) from 2004-2010 was performed. (Supplementary Table 1). A Mandard score of ≤2 indicated a pathological response in the corresponding resection specimen [37]. Total RNA was extracted using the Ambion Recoverall Kit (Thermo Fisher Scientific, Waltham, MA) and amplified using the NuGEN WT-Ovation FFPE System (NuGEN, San Carlos, CA). The amplified product was hybridized to the Almac Xcel™ Array (Almac, Craigavon, United Kingdom) and analysed using the Affymetrix 7G scanner (Affymetrix, Santa Clara, CA). Expression data is available at ArrayExpress (Accession Number E-MTAB-4666).

### Discovery and validation tissue microarrays

The discovery set of 152 FFPE OAC resection specimens (matched gene expression data available for 58 cases) were collected from 2004-2012 at the NICC. All patients received neo-adjuvant chemotherapy prior to surgical resection and had a median follow up time of 48.8 months. Pathological staging was defined according to International Union Against Cancer (UICC) TNM staging, 7^th^ edition. Eight specimens had no tumour tissue identifiable and 3 died within 3 months of surgery of causes unrelated to their cancer, resulting in 141 cases entering the final analysis (Table 1).

Validation was performed using a TMA generated from 262 OAC samples from patients who underwent potentially curative surgery at one of six Oesophageal Cancer Clinical and Molecular Stratification (OCCAMS) study group centres (Table 1). The TMAs were constructed as previously described [16, 38].

### Immunohistochemistry

A 3-μm thick section was deparaffinised and endogenous peroxidase activity was quenched with 0.3% hydrogen peroxide prior to staining using the Ventana Discovery XT^®^ automated immunostainer (Ventana Medical Systems Inc, Tuscon, AZ). Antibodies to GLUT1 (Ventana), c-MET (CONFIRM anti-Total c-MET (SP44), Ventana) and Her2 (anti-HER2/neu (4B5), Ventana) were used according to the manufacturer's instructions. Staining for p53 was performed as previously described [39]. Sections were incubated with GLUT1 antibody at 37°C for 8 minutes prior to use of the Omnimap^®^ anti-rabbit HRP conjugate detection kit (Ventana). Lung adenocarcinoma tissue was used as a positive control and test tissue with no primary antibody was used as a negative control.

GLUT1 staining was scored by three independent observers (RT, SMcQ & JJ) who were blinded to the clinical data. In cases of discordance a consensus score was reached after discussion. GLUT1 immunoreactivity was considered positive when strong homogeneous staining was observed at the cancer cell membrane or cytoplasm. Scoring was based on intensity (0 = no staining, 1 = weak, 2 = moderate and 3 = strong staining observed) with the highest intensity from the three cores used for analysis.

### Statistical analysis

Microarray data analysis was performed using Partek Genomics Suite software, version 6.6 (Partek Inc., St Louis, MO). Data was normalized using the Robust Microarray Averaging (RMA) method. Statistical analysis was performed using R (‘RMS’, ‘HMISC’, ‘COXNET’, ‘Survival’, ‘epiR’ and ‘powerSurvEpi’ packages). Gene Set Enrichment Analysis (GSEA) using the Molecular Signature Database (MSigDB v5.0) was performed as previously described [40]. Associations between GLUT1 status and the clinicopathological characteristics were calculated using chi-squared tests, with p-value adjustment for multiple comparisons using the Bonferroni correction method. Relapse-free and overall survival were calculated from the date of surgery to the date of clinical or pathological recurrence or death from any cause, respectively.

In the discovery cohort, univariate and multivariate analyses were performed using Cox proportional hazards regression, with hazard ratios and p-values reported. Proportional hazards assumptions were tested using Schoenfeld residuals. In multivariate Cox proportional hazards analysis variable selection was performed using a lasso penalty approach with cross-validation [41]. The concordance index (c-index) was used to assess the predictive fit of each model. Ranging from 0 to 1, a c-index of 0.5 suggests a model that is no better than random, while a c-index between 0.6 and 0.7 is common in survival models. Observed c-indices for each multivariate model combination were validated using bootstrap resampling validation (n = 150). Nomograms were constructed from the Cox proportional hazards models, from which prognostic indices were derived. Survival curves were estimated using the Kaplan-Meier method and compared using the log-rank test. In the validation cohort, using the variables identified in the discovery cohort, a multivariate Cox proportional hazards regression analysis (stratified by site) was performed and a prognostic index (OS) calculated and survival compared as before. Significance was set at p = 0.05, unless otherwise stated.

## SUPPLEMENTARY MATERIALS FIGURES AND TABLES


