# Supplementary material for: Glucose transporter 1 expression as a marker of prognosis in oesophageal adenocarcinoma
Source: Oncotarget. 2018 Apr 6;9(26):18518–28. doi: 10.18632/oncotarget.24906 (PMC5915089; doi:10.18632/oncotarget.24906)
Supplement: Supplementary file 1 [file oncotarget-09-18518-s001.pdf]

Glucose transporter 1 expression as a marker of prognosis in oesophageal adenocarcinoma

SUPPLEMENTARY MATERIALS

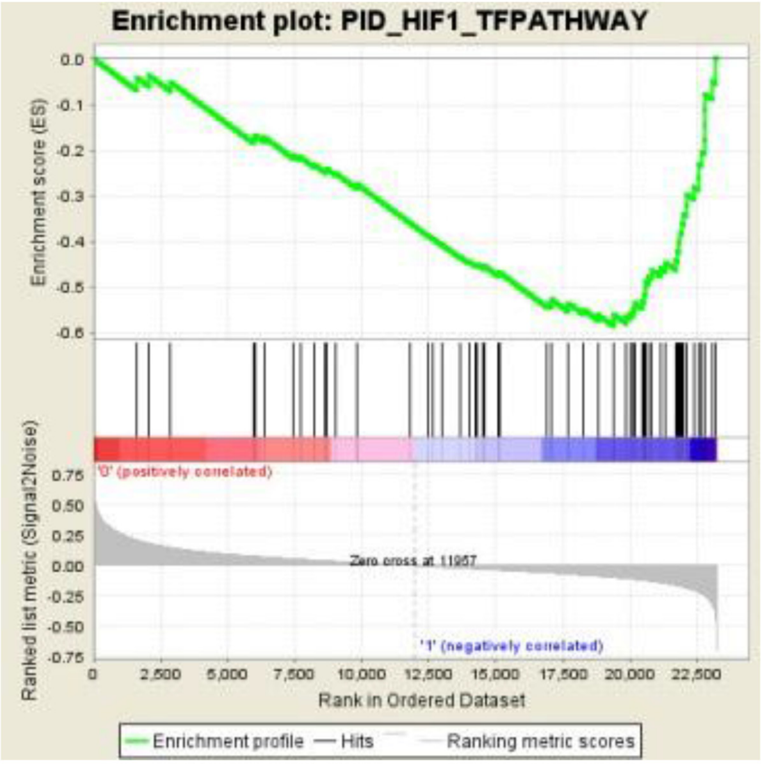

Supplementary Figure 1: Enrichment plot for the PID_HIF1_TFPATHWAY from the Canonical Pathways (C2) curated gene sets.

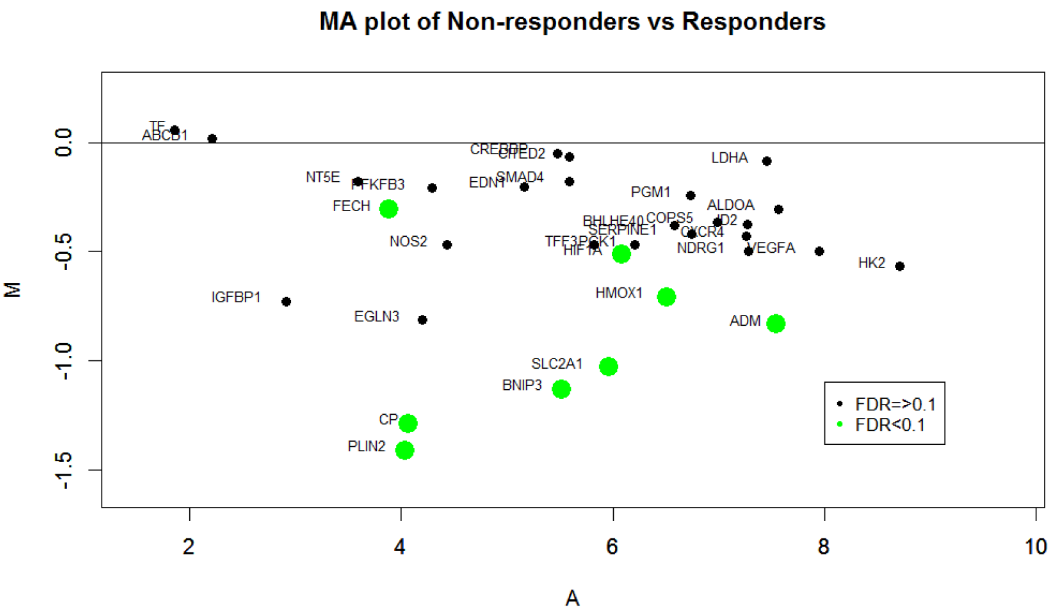

Supplementary Figure 2: MA plot of M (log ratio of expression) and A (mean of expression) of genes within the Hypoxia-Inducible Factor 1 (HIF1) pathway. The Mann-Whitney unpaired test was used to compare non-responders to responders using gene expression levels and a False discovery rate (FDR) (Benjamini and Hochberg) applied.

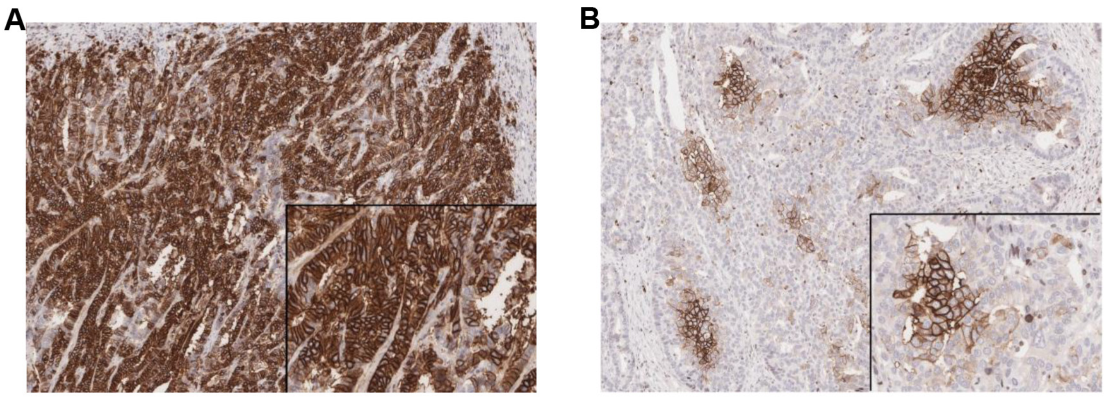

Supplementary Figure 3: Representative GLUT1 staining in oesophageal adenocarcinoma. (A) GLUT1 expression showing strong homogeneous staining (3+) at the cell membrane in tumour cells and absence of GLUT1 staining in the surrounding stroma. **(B)** EAC resection specimen demonstrating focal extent and strong intensity of GLUT1 staining. Representative 10X and 40X views are shown.

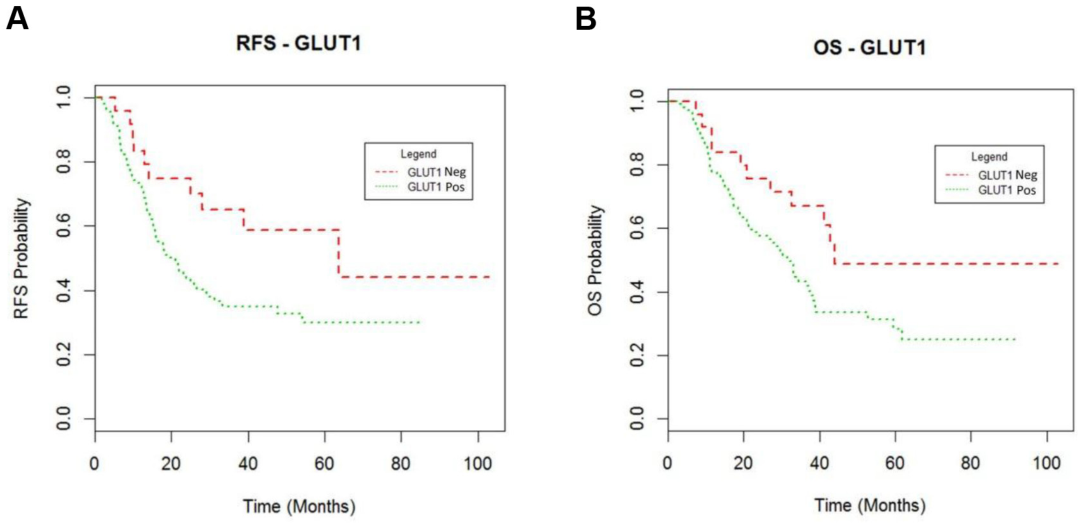

Supplementary Figure 4: Kaplan-Meier plots of relapse-free **(A)** and overall survival **(B)** comparing GLUT1 positive and GLUT1 negative patients in the discovery set.

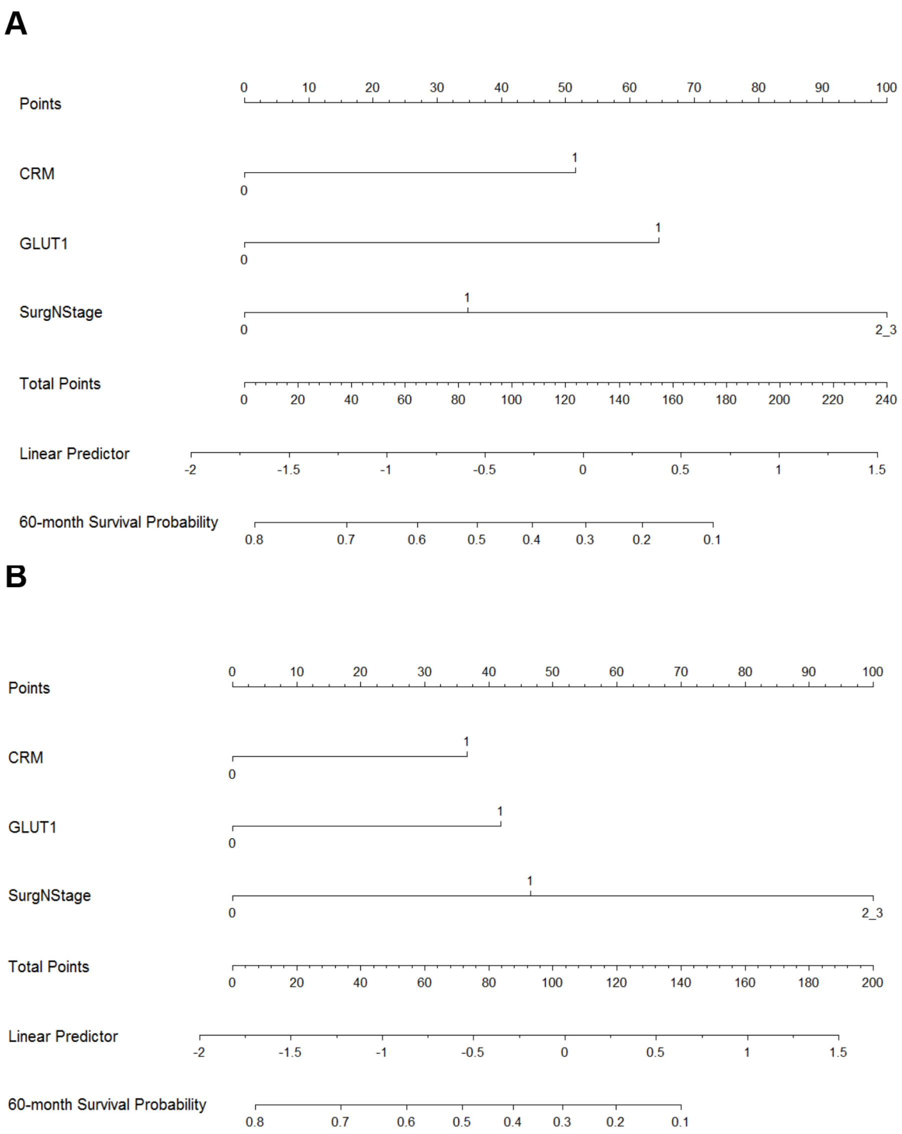

Supplementary Figure 5: Survival probability nomograms for relapse-free **(A)** and overall survival **(B)** based on the GLUT1/CRM/N stage multivariate model.

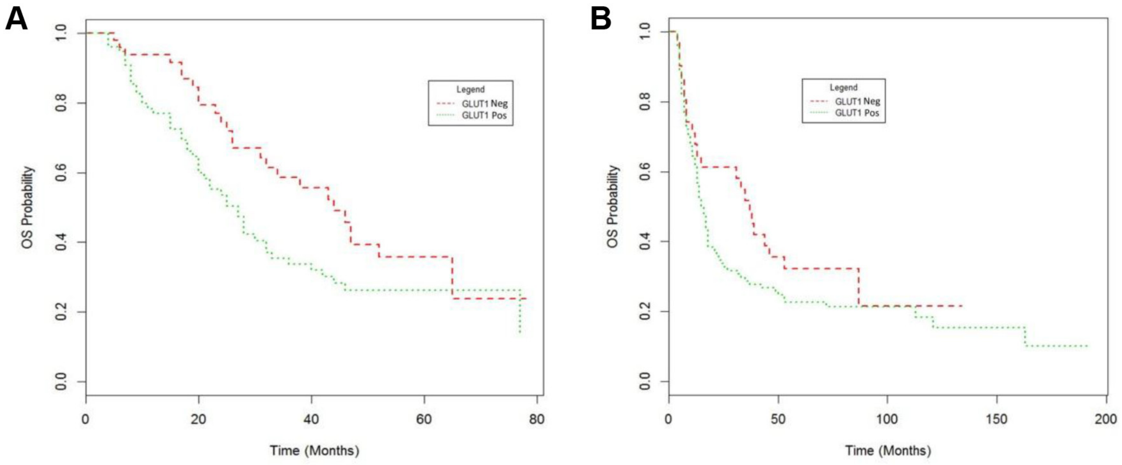

Supplementary Figure 6: Kaplan-Meier plots of comparing GLUT1 positive and GLUT1 negative patients in patients treated with NAC and surgery **(A)** and those treated with surgery alone **(B)** in the validation set.

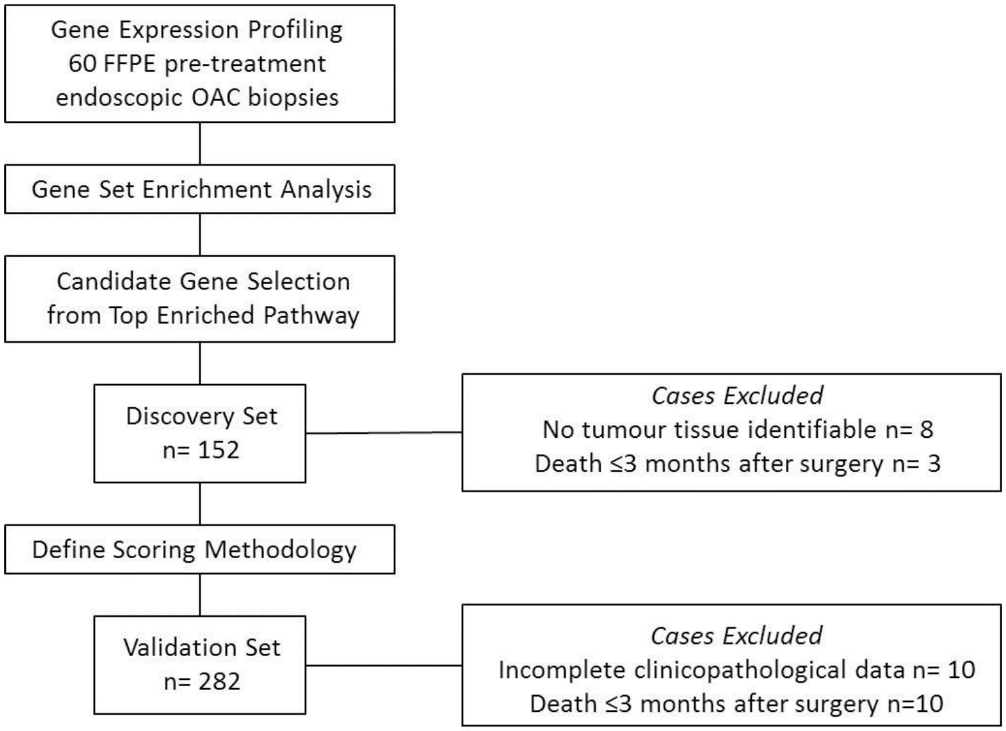

Supplementary Figure 7: CONSORT diagram.

Supplementary Table 1: Clinical and pathological characteristics of 60 FFPE pre-treatment endoscopic biopsies profiled using the Almac Diagnostics Xcel array™

	FFPE Biopsies (n=60)	
	n	%
**Age**		
<60	20	33.3
60-69	28	46.7
≥ 70	12	20
Median	62	
Range	39-78	
**Sex**		
Male	48	80
Female	12	20
**Tumour Site**		
Oesophagus	6	10
GOJ, Siewert 1	41	68.3
GOJ, Siewert 2	10	16.7
GOJ, Siewert 3	3	5
**Depth of invasion (T stage)**		
pT0/1	10	16.7
pT2	11	18.3
pT3	36	60
pT4	2	3.3
Unknown	1	1.7
**Lymph node involvement (N stage)**		
N0	23	38.3
N1	13	21.7
N2/3	23	38.3
Unknown	1	1.7
**Differentiation**		
Well	4	6.7
Moderate	24	40
Poor	30	50
Unknown	2	3.3
**Lymphovascular Invasion**		
Negative	22	36.7
Positive	36	60
Unknown	2	3.3
**Circumferential Margin Involvement**		
Negative	32	53.3
Positive	26	43.3
Unknown	2	3.3
**Neo-adjuvant chemotherapy**		
Yes	60	100
No	0	0

Supplementary Table 2: Top ten pathways enriched in pathological responders following Gene Set Enrichment Analysis with the C2 Canonical Pathways gene set

NAME	SIZE	Enrichment Score	Normalized Enrichment Score	Nominal p-value	FDR q-value	FWER p-value
PID_HIF1_TFPATHWAY	66	-0.5856576	-2.0210462	<0.0001	0.27173433	0.11
KEGG_ GLYCOSAMINOGLYCAN_ BIOSYNTHESIS_ CHONDROITIN_SULFATE	21	-0.702389	-1.9410489	<0.0001	0.37426656	0.245
BIOCARTA_MTA3_ PATHWAY	16	-0.6478256	-1.9296671	<0.0001	0.28879526	0.277
BIOCARTA_P38MAPK_ PATHWAY	39	-0.5511342	-1.9207591	0.002132196	0.24134214	0.301
PID_ILK_PATHWAY	45	-0.45857394	-1.8347195	0.025263159	0.5051053	0.51
REACTOME_MYD88_MAL_ CASCADE_INITIATED_ON_ PLASMA_MEMBRANE	80	-0.50655717	-1.8343298	0.002066116	0.4242736	0.513
REACTOME_ ELONGATION_ARREST_ AND_RECOVERY	31	-0.56351846	-1.8289493	0.00390625	0.38259107	0.527
PID_KIT_PATHWAY	52	-0.44070584	-1.8130562	0.006479482	0.39925313	0.566
KEGG_CHRONIC_ MYELOID_LEUKEMIA	73	-0.43623424	-1.8102854	0.004376368	0.36463857	0.572
PID_WNT_ NONCANONICAL_ PATHWAY	32	-0.47900793	-1.783817	0.00814664	0.4330548	0.642

Supplementary Table 3: PID_HIF1_TFPATHWAY genes enriched in the Pathological Non-responder phenotype, beginning with the most upregulated genes

GENE SYMBOL	GENE_TITLE	RANK IN GENE LIST	RANK METRIC SCORE	RUNNING ES
IGFBP1	insulin-like growth factor binding protein 1	23204	-0.42243	0.00164
PLIN2	perilipin 2	23054	-0.31181	-0.04816
ADM	adrenomedullin	22801	-0.25054	-0.0788
BNIP3	BCL2/adenovirus E1B 19kDa interacting protein 3	22793	-0.24859	-0.11184
SLC2A1 (GLUT1)	solute carrier family 2 (facilitated glucose transporter), member 1	22790	-0.24745	-0.14485
NDRG1	N-myc downstream regulated gene 1	22767	-0.24353	-0.17684
HMOX1	heme oxygenase (decycling) 1	22651	-0.22689	-0.20429
EGLN3	Egl-9 family hypoxia-inducible factor 3	22588	-0.2204	-0.23182
CP	ceruloplasmin (ferroxidase)	22566	-0.21786	-0.26025
TFF3	trefoil factor 3 (intestinal)	22371	-0.20084	-0.28088
NT5E	5’-nucleotidase, ecto (CD73)	22108	-0.18387	-0.29631
FECH	ferrochelatase (protoporphyria)	22100	-0.18339	-0.32048
NOS2	Nitric oxide synthase 4	21975	-0.17545	-0.33954
EDN1	endothelin 1	21948	-0.17406	-0.36178
BHLHE40	Basic helix-loop-helix family, member e40	21856	-0.16921	-0.38102
ALDOA	aldolase A, fructose-bisphosphate	21819	-0.16745	-0.40199
CXCR4	chemokine (C-X-C motif) receptor 4	21759	-0.16486	-0.42174
HK2	hexokinase 2	21719	-0.16278	-0.442
HIF1A	hypoxia-inducible factor 1, alpha subunit (basic helix-loop-helix transcription factor)	21338	-0.14739	-0.44727
CITED2	Cbp/p300-interacting transactivator, with Glu/Asp-rich carboxy-terminal domain, 2	21124	-0.13966	-0.4577
LDHA	lactate dehydrogenase A	20806	-0.12952	-0.46261
COPS5	COP9 constitutive photomorphogenic homolog subunit 5 (Arabidopsis)	20715	-0.1271	-0.47597
PFKFB3	6-phosphofructo-2-kinase/fructose-2,6-biphosphatase 3	20577	-0.12351	-0.48698
ABCB1	ATP-binding cassette, sub-family B (MDR/TAP), member 1	20559	-0.12316	-0.50269
TF	transferrin	20523	-0.12212	-0.51757
SMAD4	SMAD, mothers against DPP homolog 4 (Drosophila)	20472	-0.12075	-0.53167
ID2	inhibitor of DNA binding 2, dominant negative helix-loop-helix protein	20163	-0.11335	-0.53445
PGM1	phosphoglucomutase 1	20141	-0.11297	-0.54864
PGK1	phosphoglycerate kinase 1	19994	-0.10974	-0.55737
VEGFA	vascular endothelial growth factor A	19842	-0.10638	-0.56547
SERPINE1	serpin peptidase inhibitor, clade E (nexin, plasminogen activator inhibitor type 1), member 1	19385	-0.09795	-0.55995
CREBBP	CREB binding protein (Rubinstein-Taybi syndrome)	19376	-0.09775	-0.57265

Supplementary Table 4: Contingency table of GLUT1, MET, Her2 and p53 expression

Multivariate Model Option	GLUT1 Positive	GLUT1 Negative	*p* - value
**MET**			
MET Positive	94	7	0.542
MET Negative	9	1	
**Her2**			
Her2 Positive	17	4	1
Her2 Negative	97	22	
**p53**			
p53 Positive	91	20	0.587
p53 Negative	23	6	

Supplementary Table 5: Correlation of clinicopathological factors with GLUT1 expression in the Discovery (NICC) cohort

	GLUT1 Negative *n* = 27 (%)	GLUT1 Positive *n* = 114 (%)	*p*-value
**Age**			
<60	5 (19)	37 (32)	
60-69	16 (59)	51 (45)	0.302
≥ 70	6 (22)	26 (23)	
Median	64	63	0.404†
Range	47-78	28-83	
**Sex**			
Male	21 (78)	89 (78)	0.974
Female	6 (22)	25 (22)	
**ECOG Performance Status**			
0	5 (19)	37 (32)	
1	22 (81)	64 (56)	0.116
2	0	4 (4)	
Unknown	0	9 (8)	
**Tumour Site**			
Oesophagus	5 (19)	17 (15)	
GOJ, Siewert 1	14 (52)	58 (51)	0.805
GOJ, Siewert 2	5 (19)	30 (26)	
GOJ, Siewert 3	3 (11)	9 (8)	
**PET Response**			
Responder	9 (33)	34 (30)	
Non-Responder	12 (44)	51 (45)	0.914
Unknown	6 (22)	29 (25)	
**Depth of Invasion (T stage)**			
pT0/1	4 (15)	11 (10)	
pT2	4 (15)	23 (20)	0.546
pT3	19 (70)	75 (66)	
pT4	0	5 (4)	
**Lymph node Involvement (N Stage)**			
N0	14 (52)	37 (32)	
N1	4 (15)	25 (22)	0.168
N2/3	9 (33)	52 (46)	
**Differentiation**			
Well	4 (15)	2 (2)	
Moderate	6 (22)	47 (41)	0.004
Poor	17 (63)	64 (56)	
Unknown	0	1 (1)	
**Lymphovascular Invasion**			
Negative	10 (37)	37 (32)	
Positive	16 (59)	77 (68)	0.646
Unknown	1 (4)	0	
**Circumferential Margin Involvement**			
Negative	11 (41)	66 (58)	
Positive	15 (56)	48 (42)	0.191
Unknown	1 (4)	0	

†Mann-Whitney *U* Test.
ECOG – Eastern cooperative oncology group, GOJ – Gastro-esophageal junction.
PET Response defined as ≥35% reduction in Standardised Uptake Variable (SUV).

Supplementary Table 6: Univariate and multivariate analysis of clinicopathological factors, GLUT1 expression, relapse-free and overall survival in the Discovery cohort

	Relapse-free Survival			Overall Survival			Relapse-free Survival			Overall Survival		
	Hazard Ratio	95% CI	*p*-value	Hazard Ratio	95% CI	*p* -value	Hazard Ratio	95% CI	*p* -value	Hazard Ratio	95% CI	*p* -value
**CRM**												
Negative	1			1			1			1		
Positive	3.262	2.047-5.199	<0.001	3.793	2.36-6.096	<0.001	1.876	1.004-3.507	0.048	1.574	0.834-2.970	0.162
**LVI**												
Negative	1			1			1			1		
Positive	2.698	1.586-4.59	<0.001	3.816	2.128-6.844	<0.001	1.293	0.696-2.401	0.417	1.738	0.896-3.371	0.102
**T Stage**												
0,1,2	1			1			1			1		
3,4	2.503	1.442-4.343	0.0011	3.448	1.895-6.272	<0.001	1.024	0.506-2.075	0.947	1.28	0.601-2.725	0.522
**N Stage**												
0	1			1			1			1		
1	1.847	0.922-3.704	0.0837	2.482	1.175-5.245	0.017	1.374	0.618-3.055	0.436	1.548	0.661-3.626	0.315
2*	5.067	2.684-9.564	<0.001	7.085	3.611-13.9	<0.001	3.743	7.73-8.098	0.001	4.23	1.871-9.564	0.001
3*	6.12	3.225-11.612	<0.001	9.222	4.618-18.416	<0.001						
**Differentiation**												
Poor	1			1			1			1		
Moderate	0.335	0.081-1.38	0.13	0.284	0.037	0.081	1.163	0.696-1.946	0.564	1.027	0.179-3.242	0.745
Well	0.683	0.429-1.088	0.109	0.594	0.369	0.032	0.784	0.179-3.441	0.747	0.783	0.61-1.729	0.919
**GLUT1**												
Low	1			1			1			1		
High	2.069	1.091-3.925	0.026	1.85	1.009-3.39	0.047	2.106	1.056-4.201	0.035	1.895	0.973-3.691	0.06

*N2 and N3 combined for multivariate analysis due to similar hazard ratios and low patient numbers.

Supplementary Table 7: Contribution of selected variables, using elastic net penalty and cross-validation, for relapse-free and overall survival

Variable	Contribution to Model (RFS)	Contribution to Model (OS)
**Surgical N Stage**	0.665	0.757
**CRM**	0.475	0.409
**GLUT1**	0.478	0.410
**Surgical T**	0	0.102
**LVI**	0.013	0.303
**Differentiation**	0	0

Supplementary Table 8: Observed c-indices for each multivariate model combination using both observed and resampling validation for overall and relapse-free survival

Multivariate Model Option	Overall Survival		Relapse-free Survival	
	Observed	Resampling validation	Observed	Resampling validation
Surgical N Stage/ CRM/GLUT1	0.778	0.774	0.746	0.736
CRM/GLUT1	0.698	0.695	0.675	0.668
LVI/GLUT1	0.654	0.627	0.631	0.627
Surgical T/GLUT1	0.645	0.64	0.623	0.621
Surgical N/GLUT1	0.691	0.688	0.678	0.673

Supplementary Table 9: Effect of the addition of each factor to the prognostic model as assessed by log likelihood ratio

Factor added	Log likelihood	*p*-value
NULL	-342.84	
N Stage	-313.57	<0.0001
CRM	-311.32	<0.05
GLUT1	-308.36	<0.05

Supplementary Table 10: Clinicopathological factors of patients in the Validation (OCCAMS) cohort treated with chemotherapy and surgery or surgery alone

	Surgery Alone	Chemotherapy and Surgery	*p*-value
	n = 135 (%)	*n* = 127 (%)	
**Age**			
<60	30 (22)	50 (39)	
60-69	47 (35)	45 (35)	0.005
≥ 70	54 (40)	32 (25)	
Unknown	4 (3)	0	
Median	67	63	<0.001†
Range	33-88	34-80	
**Sex**			
Male	101 (75)	112 (88)	0.006
Female	34 (25)	15 (12)	
**Depth of Invasion (T stage)**			
pT0/1	8 (6)	13 (10)	
pT2	30 (22)	26 (20)	0.617
pT3	94 (70)	86 (68)	
pT4	3 (2)	2 (2)	
**Lymph node Involvement (N Stage)**			
N0	40 (30)	31 (24)	
N1	86 (64)	90 (71)	0.077
N2/3	9 (7)	2 (2)	
Unknown	0	4 (3)	
**Differentiation**			
Well	5 (4)	16 (13)	
Moderate	37 (27)	42 (33)	0.003
Poor	91 (67)	60 (47)	
Unknown	2 (1)	9 (7)	
**Lymphovascular Invasion**			
Negative	41 (30)	57 (45)	
Positive	34 (25)	67 (53)	0.234
Unknown	60 (44)	3 (2.4)	
**Circumferential Margin Involvement**			
Negative	42 (31)	91 (72)	
Positive	33 (24)	28 (22)	0.0028
Unknown	60 (44)	8 (6.3)	

†Mann-Whitney *U* Test.

Supplementary Table 11: Correlation of clinicopathological factors with GLUT1 expression in the Validation (OCCAMS) cohort

	GLUT1 Negative	GLUT1 Positive	*p*-value
	n = 80 (%)	n = 182 (%)	
**Age**			
<60	24 (30)	56 (31)	
60-69	29 (36)	63 (35)	0.937
≥ 70	25 (31)	61 (34)	
Unknown	2 (3)	2 (1)	
Median	66	66	0.852[†]
Range	40-81	33-88	
**Sex**			
Male	64 (80)	149 (82)	0.721
Female	16 (20)	33 (18)	
**Depth of Invasion (T stage)**			
pT0/1	8 (10)	13 (7)	
pT2	20 (25)	36 (20)	0.296
pT3	52 (65)	128 (70)	
pT4	0	5 (3)	
**Lymph node Involvement (N Stage)**			
N0	21 (26)	51 (28)	
N1	22 (28)	70 (38)	0.089
N2/3	37 (46)	59 (32)	
Unknown	0	2 (1)	
**Differentiation**			
Well	8 (10)	13 (7)	
Moderate	21 (26)	58 (32)	0.574
Poor	46 (58)	105 (58)	
Unknown	5 (6)	6 (3)	
**Lymphovascular Invasion**			
Negative	38 (48)	60 (33)	
Positive	40 (50)	61 (34)	0.905
Unknown	2 (3)	61 (34)	
**Circumferential Margin Involvement**			
Negative	55 (69)	78 (43)	
Positive	25 (31)	36 (20)	0.961
Unknown	0	68 (37)	
**Neo-Adjuvant chemotherapy**			
Yes	49 (61)	78 (43)	0.006
No	31 (39)	104 (57)	

[†]Mann-Whitney *U* Test.
ECOG – Eastern cooperative oncology group, GOJ – Gastro-esophageal junction.
PET Response defined as ≥35% reduction in Standardised Uptake Variable (SUV).

Supplementary Table 12: Multivariate analysis of the Validation cohort patients treated by surgery alone/ chemotherapy and surgery

	Surgery Alone (*n* = 135)			Chemotherapy and Surgery (*n* = 127)		
	**Hazard Ratio**	**95% CI**	***p*-value**	**Hazard Ratio**	**95% CI**	***p*-value**
**CRM**						
Negative	1			1		
Positive	0.753	0.390-1.455	0.399	1.401	0.747-2.63	0.293
**N Stage**						
0	1			1		
1	6.427	2.292-18.024	<0.001	1.671	0.68-4.104	0.263
2, 3	11.55	4.018-33.202	<0.001	5.525	2.532-12.057	<0.001
**GLUT1**						
Low	1			1		
High	1.26	0.706-2.24	0.436	1.704	1.014-2.862	0.0442

Supplementary Table 13: Prognostic model incorporating N stage, CRM and GLUT1 in the Validation cohort

**Prognostic Group**	**Surgery Alone (*n* = 135)**	**Chemotherapy and Surgery (*n* = 127)**
	**Median OS (95% CI months)**	**Median OS (95% CI months)**
**Group 1**	Not reached	65 (47-NA)
**Group 2**	33 (13-NA)	34 (20-NA)
**Group 3**	15 (11-21)	20 (18-26)

Supplementary Table 14: Comparison of the reporting of GLUT1 as a prognostic marker in oesophageal adenocarcinoma with the REMARK guidelines

REMARK Guidelines Criteria	GLUT1 Project
**Introduction**	
State the marker examined, study objectives and pre-specified hypothesis	Identify candidate prognostic markers through the analysis of whole transcriptome data and select a marker for further study. Define a scoring methodology for this marker in a discovery set and validate it in an independent set of patients.<br>Immunohistochemistry for Glucose Transporter 1 (GLUT1), examine the prognostic ability of GLUT1 alone and in combination with other clinicopathological factors.
**Materials and Methods**	
Patients	
Describe the characteristics	Discovery cohort(n=141) and Validation set (n=262). See Table 1.
Describe the treatments received and how chosen	Localized oesophageal and gastro-oesophageal junction adenocarcinomas treated with surgical resection +/- neo-adjuvant chemotherapy
Specimen Characteristics	
Type of biological material used, methods of preservation and storage	Formalin fixed paraffin embedded (FFPE) whole face sections and Tissue Microarrays (TMAs).
Assay Methods	
Specify assay used and provide a detailed assay protocol	GLUT1 immunohistochemical staining (see Methods section)
Study Design	
Method of case selection, time period, end of follow-up period and median follow-up time	See Methods section
Precisely define all clinical endpoints	See Statistical Analysis section
List all candidate variables initially examined or considered for inclusion in models	T stage, N Stage, CRM status, Lymphovascular invasion, GLUT1
Give rationale for sample size	Initially the event per variable (EPV) guideline was applied to the discovery cohort, i.e. for every variable introduced there should be at least ten events. This was exceeded in the discovery cohort (EPV=16) given the initial variables considered, with 80 events (in both OS and RFS). Power calculations were carried out with respect to the three variable multivariate model in both discovery and validation cohorts, based on relative ratios of each patient sub-group, number of events between groups, observed multivariate hazard ratios and an alpha of 0.05.

**Discovery**	**Power**	
CRM	75.9%	
GLUT1	88.3%	
Surgical N	42.8%/99.6%	
**Validation (surgery)**	**Power**	**Validation (Surgery/Chemo)**
CRM	17.6%	18.9%
GLUT1	18.5%	63.6%
Surgical N	98.4%/99.9%	24.7%/99.9%

Statistical Analysis Methods	
Specify all statistical methods	See Methods section

(Continued)

	Clarify how marker values were handled and describe methods used for cutpoint determination	Cutpoints were determined using the Pathology Integromics in Cancer (PICan) system. For simplicity of scoring a cut point of any cells staining for GLUT1 were taken as positive.
**Results**		
	Data	
	Describe the flow of patients and reasons for dropout	Clinicopathological characteristics of the Discovery and Validation sets are described in Table 1 and the flow of patients in Figure S1
	Report distributions of basic demographic characteristics	See Tables 1, S5 and S8
	Analysis and presentation	
	Show the relation of the marker to standard prognostic variables	Univariate and multivariate analyses for the Discovery and Validation sets are reported in Tables 3, 4 and Table S5 & S6
	Present univariate analyses for all other variables	See Table S6
	For multivariate analyses report estimated effects with confidence intervals	See Table 3
	Provide estimated effects from an analysis in which the marker and standard prognostic variables are included	See Results section
	Report results of further investigations	
**Discussion**		
	Interpret the results in the context of the pre-specified hypothesis, other relevant studies and limitations	See Discussion
	Discuss implications for future research and clinical value	
